# Women’s experiences of two different self-assessment methods for monitoring fetal movements in full-term pregnancy - a crossover trial

**DOI:** 10.1186/1471-2393-14-349

**Published:** 2014-10-07

**Authors:** Mari-Cristin Malm, Ingela Rådestad, Christine Rubertsson, Ingegerd Hildingsson, Helena Lindgren

**Affiliations:** School of Health and Social Studies, Dalarna University, 791 88 Falun, Sweden; Department of Women’s and Children’s Health, Uppsala University, 751 85 Uppsala, Sweden; Sophiahemmet University, Box 5605, 114 86 Stockholm, Sweden; Department of Women’s and Children’s Health, Karolinska Institutet, 171 77 Stockholm, Sweden; Department of Nursing, Mid Sweden University, 85170 Sundsvall, Sweden; Department of Health and Caring Sciences, Sahlgrenska Academy University of Gothenburg, Gothenburg, Sweden

**Keywords:** Self-assessment, Fetal movements, Pregnancy, Crossover trial

## Abstract

**Background:**

Low maternal awareness of fetal movements is associated with negative birth outcomes. Knowledge regarding pregnant women’s compliance with programs of systematic self-assessment of fetal movements is needed. The aim of this study was to investigate women’s experiences using two different self-assessment methods for monitoring fetal movements and to determine if the women had a preference for one or the other method.

**Methods:**

Data were collected by a crossover trial; 40 healthy women with an uncomplicated full-term pregnancy counted the fetal movements according to a *Count-to-ten* method and assessed the character of the movements according to the *Mindfetalness* method. Each self-assessment was observed by a midwife and followed by a questionnaire. A total of 80 self-assessments was performed; 40 with each method.

**Results:**

Of the 40 women, only one did not find at least one method suitable. Twenty of the total of 39 reported a preference, 15 for the Mindfetalness method and five for the Count-to-ten method. All 39 said they felt calm, relaxed, mentally present and focused during the observations. Furthermore, the women described the observation of the movements as safe and reassuring and a moment for communication with their unborn baby.

**Conclusions:**

In the 80 assessments all but one of the women found one or both methods suitable for self-assessment of fetal movements and they felt comfortable during the assessments. More women preferred the Mindfetalness method compared to the count-to-ten method, than vice versa.

## Background

The movements of the fetus are unique for every fetus, as are also the mother’s experiences of these movements. The frequency of movements increases from week 24 of pregnancy until week 32. From week 32 on, the frequency of fetal movements tends to plateau until the onset of labor, thus the frequency of fetal movement does not normally decrease at the end of pregnancy [1]. However, the type of movements may change as the pregnancy advances [2].

Women’s ability to perceive fetal movements is affected by factors such as gestational age, parity, obesity and the localization of the placenta [1, 3]. The greatest frequency of fetal movements is experienced when the women are lying down and a majority of women perceive most movements in late evening [4, 5].

Maternal concerns about fetal movements are a common reason for unscheduled antenatal visits. In different populations between four and 15 percent of pregnant women will contact health care providers with concerns for the fetal activity [6].

Methods for systematically counting and documenting fetal movements as a measure of the wellbeing of the fetus have been used in the care of pregnant women for more than 40 years. The counting methods can be divided into two main groups: 1) Measuring the time it takes to observe a specified number of movements, 2) Counting the number of movements identified during a specified time [6].

The first method, the *Count-to-ten* method, was introduced by Pearson and Weaver in 1976. The method instructs the care-giver to encourage the mother-to-be to measure the time it takes to feel 10 movements [7]. In 1973, Sadovsky and Yaffe introduced the second method, counting the number of fetal movements in a specified time interval; for example the number of movements in a ten-minutes period [8].

It is not only the number of movements per unit time that may be of importance but also the strength and/or nature of those movements. To take this into account, *a method for observing the character of movement* was introduced by Rådestad in 2012, who gave this method the name *Mindfetalness*, a name given to suggest an association with the practice of Mindfulness. This method requires that the mother-to-be should be encouraged to lie on her left side during a period of her baby’s wakefulness and during 15 minutes focus on exactly how the baby is moving taking note of the strength, type and frequency of movement, but not counting the number of separate movements per unit time [9].

Saastad et al. found that low maternal awareness of fetal movements is associated with women giving birth to babies small for gestation age [10]. Nijhuis [11], and Vindla and James [12] suggest that development of appropriate methods to analyze behavior of the fetus should have high priority in clinical research. Raynes-Greenow et al. suggested that women’s own assessments of fetal movements be used in antenatal care as a possible indicator of fetal wellbeing [13].

A Cochrane Review published in 2007 emphasized the need for further research designed to define optimal screening and investigation for women perceiving decreased fetal movements and also to help women evaluate the possibility of using different methods for observing fetal movements [14].

The aim of this study was to investigate women’s experiences of and preferences for two self-assessment methods for monitoring and measuring two different aspects of fetal movements.

## Methods

This is a crossover trial including women in full-term pregnancy where women were instructed to use two different methods to assess fetal movements 1: Count the time it takes to feel 10 fetal movements according to a *Count-to-ten* method, modified by Frøen et al. [15] and 2: Observe the character and frequency of the movements instead of counting fetal movements, according to the *Mindfetalness* method [9].

### Recruitment and participants

Data collection was carried out in Dalarna county in the middle of Sweden, from July 4, 2013 until January 24, 2014.

For the purpose of the study a consecutive sample of 40 women was decided. All 23 midwives in a total of six antenatal clinics within the area were asked to provide information about the study to pregnant women enrolled at their clinics during the study period. Inclusion criteria for the study were being a primipara, having a full-term singleton pregnancy, and following the standard visiting schedule for antenatal care. Excluded were women with a high-risk pregnancy or complications, and women who had not mastered the Swedish language. Recruitment stopped after a number of 40 women had performed two self-assessments of fetal movements. In total 78 women were approached of whom 38 did not participate in the study (Table 1).

**Table 1:** **Women not participating in the study (N = 38)**

Reason	n	Explanation
Not possible to contact	15	Did not answer telephone call and did not decline by sending reply form
Declined by reply form	1	No explanation given
Declined by telephone	5	*“stressed out”*
		*“will soon give birth”*
		*“difficult to explain how fetal movements feels”*
		*“not that interested, not feeling well”*
		*“having a planned caesarean section”*
		Gave birth before the first observation
Gave birth	5	No explanation given
Canceled planned observation	8	
Midwife did not distribute letter	2	The women declines to receive written information about the study

Interrupted observation 2 Inactive baby during first observation.

The midwives at the antenatal clinics gave a brief verbal presentation about the study to the women who met the inclusion criteria and then gave them printed information. Women who agreed to participate received a reply form from their midwives and were asked to sign an informed-consent form and indicate their telephone number. The informed-consent form was then forwarded to the research team.

One woman declined to participate by sending the reply form; an additional five women declined participation by telephone, declaring reasons such as lack of motivation and time. Thirteen women who agreed to participate did not perform any self-assessment; five women gave birth before the first planned assessment and eight women canceled the planned assessment. In two cases the woman did not want to receive the written information about the study from the midwife and in an additional two cases the assessment could not be performed because the babies were not in an active phase when the researcher (M-C M) and the woman met for the first assessment (Table 1).

All women who agreed to participate in the study were asked when they usually felt the babies’ movements during daytime and an appointment for the first self-assessment was made by taking this information into account. The women chose a location convenient for them: 29 observations were carried out at the workplace of the researcher at the university in a room intended for rest, 23 in the women’s homes, 19 at an antenatal clinic and nine self-assessments were carried out in the university clinical education center at the local hospital.

### Data collection

The first 20 participants used the *Count-to-ten* method at the time for the first assessment and the *Mindfetalness* method at the second assessment. The next 20 participants used the *Mindfetalness* method at the first assessment and *Count-to-ten* at the second i.e. a crossover design was used for the data collection.

The self-assessment started after brief additional verbal information was provided by the researcher about the procedure and after being assured of an active period by the baby. The women were asked to lie down on their left side and were encouraged to change position during the assessment if they preferred. Furthermore the women were asked to speak freely and share their experiences during the assessment, recorded by a digital voice recorder.

A timer was started when the woman gave a sign that she was ready to start her self-assessment. The researcher was sitting quietly a few meters away, with the participant within sight, writing short notes every minute regarding the woman’s behavior such as change of position, facial and verbal expressions as well as body language. When the *Count-to-ten* method was used, the researcher turned off the timer immediately after the woman had noticed movement number 10. When the *Mindfetalness* method was used, the timer was stopped at 15 minutes.

After each assessment the women were asked to answer a study-specific questionnaire. The questionnaire was developed on the basis of experience in a previous study for which we used an open-ended question to a larger population. We identified emotions related to the awareness of fetal movements experienced by women in late pregnancy and questions were asked regarding the experience of the defined emotions in the present study [16]. The questionnaire included seven types of predefined emotions that the women might have experienced during the self-assessment: *calm, worried, relaxed, stressed out, mentally present, focused* and *tensed.* The alternatives for response were: *Fully agree, partly agree* or *completely disagree*. The women were asked once which method they preferred and this was done after the second assessment. Both fixed and open-ended questions were included, answering them took the women between 5 to 10 minutes to complete. The questionnaires were handed over to the researcher in a closed envelope.

#### Data analysis

The analyses were conducted using IBM® SPSS® software version 20.0 (IBM Corp., Chicago, IL, USA). Descriptive statistics were applied for presenting the study sample and Fisher’s exact test was used for analyzing Relative risk with a 95% confidence interval. The emotions were measured by a Likert scale that was dichotomized and the cut-offs were set between “completely agree” and “partly agree” or “disagree”. The observational notes were worked through using thematic analysis with presence-absence coding [17].

The Regional Research Ethic Review Board in Uppsala, Sweden approved the study (Ref No 2013: 092) and informed consent was obtained from all participants.

## Results

A total of 40 healthy women in gestational weeks 37–39 who did not report any ongoing complication during pregnancy participated in the study. The age of the participants ranged between 21–38 years and the mean was 31.5 years.

Each woman participated in two separate self-assessments i.e. a total of 80 assessments was performed within this crossover trial. The time between the two separate occasions varied from one to 10 days (mean two days).

Twenty-six of the women were in gestational week 37, 13 were in gestational week 38 and one woman was in gestational week 39, during the first assessment. During the second assessment 20 of the women were in gestational week 37, 19 in gestational week 38 and one woman was in gestational week 39.

The time elapsed for the women to notice 10 movements in the Count-to-ten method varied from one and a half minute up to 21 minutes, the median time was eight minutes and the mean was nine minutes 52 seconds.

### Emotions during the self-assessment

In the 80 assessments none of the women indicated “disagree” with the following statements: “During the assessment I felt”: 1) *calm*, 2) *relaxed*, 3) *mentally present,* 4) *focused*. When comparing those who “agreed completely” with those who “agreed partly”, regarding the four above mentioned emotions, no statistically significant differences were found between the methods (Table 2).

**Table 2:** **Women’s experiences of emotions during systematic self –assessment of fetal movements**

Emotion I felt…	Method	Completely agree	Partlyagree	Disagree	Missing N	Completely agree RR	Partly agree
		N (%)	N (%)	N (%)			95% CI
**Calm**	Count-to-ten	36 (92)	3 (8)	0	1	1.1	0.5-9.1 ref
			6 (15)		0		
	Mindfetalness	34 (85)	9 (11)	0	1		
	Total	70 (89)		0			
**Worried**	Count-to-ten	1 (3)	0	38 (97)	1	1.0	0.9-1.1 ref
		0	1 (3)	38 (97)	1		
	Mindfetalness	1 (1)	1 (1)	76 (98)	2		
	Total						
**Relaxed**	Count-to-ten	26 (67)	13 (33)	0	1	1.0	0.4-2.8 ref
			14 (36)	0	1		
	Mindfetalness	25 (64)	27 (35)	0	2		
		51 (65)					
	Total						
**Stressed out**	Count-to-ten	0	7 (18)	33 (82)	0	1.0	0.8-1.2 ref
			5 (13)	34 (85)	0		
	Mindfetalness	1 (2)		67 (84)	0		
		1 (1)	12 (15)				
	Total						
**Present**	Count-to-ten	34 (85)	6 (15)	0	0	0.9	0.8-1.0 ref
		38 (95)	2 (5)	0	0		
	Mindfetalness	72 (90)	8 (10)	0	0		
	Total						
**Focused**	Count-to-ten	32 (80)	8 (20)	0	0	0.9	0.9-1.1 ref
		36 (90)	4 (10)	0	0		
	Mindfetalness	68 (85)	12 (15)	0	0		
	Total						
**Tense**	Count-to-ten	0	7 (18)	33 (82)	0	1.2	1.0-1.6 ref
			13 (33)	26 (67)	1		
	Mindfetalness	0	20 (25)	59 (75)	1		
	Total	0					

When comparing those who indicated “Disagree” with those who “Agree partly” and “Agree completely” regarding the measured emotions: *calm, worried, relaxed, stressed out, mentally present, focused* and *tensed*, no statistically significant differences were found between the methods (Table 2).

One woman reported that she completely agreed with the statement “during the assessment I felt worried” when she performed the *Count-to-ten method.* This woman took the longest time (21 minutes) to perceive 10 movements. The same woman completely disagreed with the statement “During the observation I felt worried” when she performed the *Mindfetalness* method.

Another woman reported that she completely agreed with the statement “during the assessment I felt stressed out” after she had performed the *Mindfetalness* method. At the same time she completely agreed with the four statements “I felt calm, relaxed, mentally present and focused” and completely disagreed with the statement “I felt worried” and “I felt tense”. The same woman partially agreed with the statement “During the assessment I felt stressed out” when performing the *Count-to-ten method*.

### Preference of method

Of the women 39 of 40 (98%) reported that one or both methods - Count-to-ten and Mindfetalness - were suitable for them. Only one woman (2%) stated that none of the methods were suitable for her RR 39.0 (95% CI 5.6-270). Of those women (n = 20) who preferred one of the methods, 15 (75%) women preferred the Mindfetalness method and five (25%) women preferred the Count-to-ten method RR 3.0 (95% CI 1.3-6.7) (Table 3).

**Table 3:** Women’s preference of self-assessment method* (N = 40)

	Prefer count-to ten	Prefer mindfetalness	Both methods are suitable	None of the methods are suitable
	n	n	n	n
**Group 1, started with the Count–to- ten method n = 20**	1	11	8	0
**Group 2, started with the Mindfetalness method n = 20**	4	4	11	1
**Total**	5	15	19	1

*The women were asked about preference of method once, after the second i.e. the last observation.

The woman who considered that none of the methods were suitable gave her reason for this evaluation:*” Unfortunately, I think my baby did not demonstrate its true self. Usually I experience my baby as much more active”* (woman No 34)

### Researcher observations

It was noticed by the researcher that during 27 of the 80 assessments (12 C*ount-to-ten* and 15 *Mindfetalness*) the woman changed her body position once or twice up to four times. The women changed from lateral left side position into semi-sitting, lateral right position or supine position. One woman preferred upright sitting during her two assessments. During 33 assessments the women remained still while during the rest of the 80 assessments (n = 47) it was observed that the women made minor body adjustments during the assessment.

During 25 of the assessments (15 *Count-to-ten* and 10 *Mindfetalness*) the women caressed the belly gentle while during the remaining 55 (25 *Count-to-ten* and 30 *Mindfetalness*) the women kept their hands at rest, often with one hand placed on the fundus of the uterus and the other hand on the lower abdomen. Furthermore it was noticed that during the assessments the women touched the belly lightly with fingertips and during four assessments the women lightly squeezed different parts of the belly.

The women kept their eyes closed all or most of the time during 25 assessments (12 *Count-to-ten* and 13 *Mindfetalness)* while during the other 55 the women had their eyes open and moved their gaze. In 16 of 80 assessments (9 *Count-to-ten and* 7 *Mindfetalness*) the researcher noticed that the women smiled and during 15 assessments (7 *Count-to-ten* and 8 *Mindfetalness*) occasionally laughed.

Fourteen women described how they experienced the movements during the self-assessment. Three women described their experience after having performed the Count-to-ten method as a positive experience during which they were in contact with the unborn baby. Eleven women described their experience after having used the *Mindfetalness* method as safe and reassuring and a moment for communication with the unborn baby (Table 4).

**Table 4:** **Women’s experience of fetal movements during the observations as expressed in their written statements**

Number reporting the experience	Number of the observation	Method	Statement
2	First	Count-to-ten	It’s satisfying and fun to feel the movements.
4	Second	Count-to-ten	It always felt good when you felt the movements.
27	First	Count-to-ten	I experienced it as the baby responded.
2	Second	Mindfetalness	Exciting to wait for the next sensation. Funny every time I feel something. Particularly stimulating if it is powerful and strong, and when you see the movements from outside. Feels safe with activity.
4	Second	Mindfetalness	It felt good all the time. It's a great feeling when you know that the baby is moving and I´m very happy and pleased.
8	Second	Mindfetalness	The movements from the baby make me calm and secure.
11	Second	Mindfetalness	Truly felt like I had contact with the baby.
12	Second	Mindfetalness	I experienced that when I was listening inward the baby was listening vis-a-vis as if there was a communication between us.
15	Second	Mindfetalness	Felt like the baby was moving even more, although I did not feel anything.
20	Second	Mindfetalness	Probably she was a little tired today, because I am.
27	Second	Mindfetalness	Once more I felt that the baby reacted when I put my hand there (on the belly)
28	Second	Mindfetalness	Lovely.
33	Second	Mindfetalness	Hard to describe.
36	Second	Mindfetalness	To feel the movements I had to concentrate more than the last time.

## Discussion

We found that almost all women in this study considered that both the Count-to-ten method and the Mindfetalness method were suitable for self-assessment of fetal movements. Among women who reported that they preferred one of the methods over the other, a majority chose the Mindfetalness method. In general the women reported that they had positive emotions during their assessment and the researcher noticed that the mothers-to-be seemed relaxed and focused during their self-assessments. The women described the assessment of the movements as safe and reassuring and a moment for communication with the unborn baby.

The women in this study stated that self-assessment of fetal movement was suitable for them. It has previously been observed that there is in general a high acceptability for systematic self-assessment of fetal movements among expectant mothers [18, 19]. In a large randomized trial which included 68 000 women the compliance was also high regarding the counting of fetal movements, 81 percent of the women in the intervention group followed the chart for counting of fetal movements [20]. The most likely explanation of this high compliance among women probably has to do with a generally high awareness of importance of fetal activity among pregnant women. Comparisons between self-assessment methods have rarely been conducted previously. Freda et al. [21] compared the *Count-to-ten* method with the *Sadovsky* method. Eighty percent of the women stated that they liked counting fetal movements and thought it was easy to do; no statistically significant differences were found in a comparison of the extent of compliance by users of the two methods. Velasquez [22] suggested that compliance is high among women who understand the rationale for fetal monitoring, have been informed about the procedure, and know that it takes no more than 1–2 hours/day. Berndl et al. [23] reported that no more than 18 percent of the 304 women included in a descriptive study had knowledge about fetal monitoring and normal fetal movement. To our knowledge no previous comparison between a self-assessment method focusing on qualitative variables and a counting method for measuring the fetal movements has been carried out. In our study almost all women reported that both methods were suitable for them and of those who preferred one method a majority preferred the qualitative method i.e. the *mindfetalness* method in which the number of movements is not determined. We can only speculate about the reason for this; one explanation could be that women focus on the unborn baby as an individual and not only the kicks to be counted. It is possible that counting is considered more prestigious to the pregnant women and is therefore not preferred to the same extent. Draper et al. [24] found that some women felt anxiety until the requisite ten kicks had been charted.

In general, the women in our study reported positive emotions during both methods for self-assessment of fetal movements. Most previous studies have reported that women do have positive experiences of the assessment of fetal movements [19, 25–27]. However, contrary to these findings a study from Draper et al. [24] suggested that the assessment might cause anxiety and worry about the baby’s condition, some expressed worry since they were not sure what was considered to be a “kick” and some women felt anxious about the timing of the fetal activity. Lack of information about the usefulness of monitoring was considered a major problem in the study. Nevertheless, it has previously been shown that maternal awareness towards the unborn baby’s health positively influences the mother–infant relationship [28, 29].

The women described the observation of the movements as safe and reassuring and a moment for communication with the unborn baby. Mikhail and co-authors [27] also reported that counting fetal movements was experienced as a possibility for connection with the unborn baby and the counting enhanced the maternal attachment to the fetus. In contrast, Saastad and co-authors [19] evaluated the effect of formally counting fetal movements (n = 473 women) and found no differences in maternal-fetal attachment scores between women who formally counted movements and those who did not. Studies focusing on the effect on attachment have used the quantitative method for assessment; counting of movements. To our knowledge, no previous studies have measured the effect on maternal attachment using a qualitative method for assessment of the fetal activity. It is possible that the concentration on quality of movements, instead of quantity, increases the opportunity to connect with the fetus.

In our study the mean time for counting to ten movements was nine minutes and 52 seconds. The assessment was performed in a period when the baby was awake but according to the participating women the baby had periods at night when they were more active. Previous studies have reported the time to count to ten movements to be less than 10 minutes [15] and approximately 10 minutes in normal pregnancies [30]. Our results corresponds well with the findings above, although several women commented that their unborn babies had more active periods later at night and that the chosen time might not have been optimal.

The Royal College of Obstetricians and Gynecologists [1] recommends that women should be advised to be aware of their baby’s individual pattern of movements and that the fetal movements should be assessed by subjective maternal perception of fetal movements. Furthermore, they state that clinicians should be aware that instructing women to monitor fetal movements is potentially associated with increased maternal anxiety. On the other hand, studies by Saastad et al. indicated that low awareness of the fetal movements is associated with adverse neonatal outcomes such as low birth weight. We cannot neglect the fact that women in our study reported that the assessment was comfortable and gave them positive emotions. Since previous studies have focused on quantitative self-assessment methods one can speculate whether a qualitative method for maternal awareness would cause less anxiety among the women. Further studies are needed in order to evaluate the benefits of systematic self-assessment for the fetal well-being among women with uncomplicated pregnancies. Systematic self-screening may also have benefits such as increased attachment and communication between the mother and child as long as they are not demanding. It is also possible that a higher level of awareness makes women feel safe and in contact with their baby, i.e. they will be less inclined to attend for unscheduled visits due to worry about decreased fetal movements if they know the baby’s usual activity and pattern. Monitoring may also prevent pre-hospital delay in those cases where decreased fetal movements are a reality, i.e. when the placenta is not functioning appropriately.

### Strengths and limitations

Our study was undertaken in only one county in Sweden and has a relatively small sample size of first time mothers, factors that limit generalizability. Data were collected with a crossover design and by triangulation; the experiences of fetal movements were reported by the women in their own words and confirmed by questionnaires and the researcher’s observation, which could strengthen the reliability of the study. Based on the midwives’ descriptions of the recruitment process we have no reason to believe that the women were selected for inclusion. The participants were aware of the study purpose and may have been affected by the observer’s presence during the observations. In light of the detailed descriptions reported by the women and the non-stressful emotions that were observed in the study, we do not believe that being observed had a particular effect. However, we cannot exclude the potential difference between participants and those who declined participation regarding the experienced negative emotions during the observations. It is possible that women who did not want to participate did so because of stressful emotions related to the situation. Our study is the first of which we are aware that accurately observed women performing self-assessment of fetal movements. Differences in the timing of counting movements may have been influenced by several factors, such as differences in the women’ s ability to focus on the movements during the observations and the women’s different preferences of method. For ethical reasons, the chosen time and place for observation was not the most optimal for the activity of the baby. Most women report that their baby is most active before they go to sleep but we did not consider bedside observations appropriate in this study. The effectiveness of the self-assessment methods for identification of foetuses at risk or the compliance of the self-assessment methods requires larger study populations and other methods. Nevertheless, the results of this study support the assumption that women feel comfortable when focusing on the movements on their baby.

## Conclusion

All but one of the studied women found one or both methods suitable for self-assessment of fetal movements. More women preferred the Mindfetalness method before the count-to-ten method, than vice versa.
